# Hypoxia-inducible factor-2α promotes tumor progression and has crosstalk with Wnt/β-catenin signaling in pancreatic cancer

**DOI:** 10.1186/s12943-017-0689-5

**Published:** 2017-07-14

**Authors:** Qi Zhang, Yu Lou, Jingying Zhang, Qihan Fu, Tao Wei, Xu Sun, Qi Chen, Jiaqi Yang, Xueli Bai, Tingbo Liang

**Affiliations:** 1 Department of Hepatobiliary and Pancreatic Surgery, the Second Affiliated Hospital, Zhejiang University School of Medicine, No. 88 Jiefang Road, Hangzhou, 310009 China; 2 Key Laboratory of Pancreatic Diseases of Zhejiang Province, Hangzhou, China; 3 Department of General Surgery, Huzhou Central Hospital, Huzhou, China

**Keywords:** EPAS1, PDAC, Protein interaction, Cancer stem cell, Metabolic shift

## Abstract

**Background:**

Pancreatic cancer is a devastating disease that is characterized by persistent hypoxia. The roles of hypoxia-inducible factor-2α (hif-2α) are different to those of hif-1α, although both are critical for tumor cells to adapt to the hypoxic microenvironment. However, unlike the well-studied hif-1α, the role of hif-2α in tumors, including pancreatic cancer, is poorly understood.

**Methods:**

Herein, we used a mutated hif-2α (A530T) to figure out the problem that wild-type hif-2α is quickly degraded which limits the study of its function. Using several cell lines, mouse models, and human tissues, we obtained a general picture of hif-2α in pancreatic cancer progression.

**Results:**

Functional assays revealed that hif-2α promotes epithelial-to-mesenchymal transition, enhances tumor proliferation and invasion, increases stemness, facilitates angiogenesis, and up-regulates aerobic glycolysis. We identified an interaction between hif-2α and β-catenin, and found that hif-2α/β-catenin complex formation increased the activity of β-catenin and the protein stability of hif-2α. In vivo study confirmed the pro-oncogenic role of hif-2α, whose expression correlated with those of E-cadherin, vimentin, Ki-67, and CD31, but not hif-1α. A human tissue study showed that hif-2α was associated with lymph node metastasis, pathological grade, stroma abundance, vascularization and patient survival. High expression of hif-2α was also identified as an independent indicator of poor prognosis in patients with pancreatic cancer.

**Conclusions:**

Our systematic study revealed the roles of hif-2α in pancreatic cancer, and may provide a novel target for this highly malignant disease.

## Background

Pancreatic cancer is one of the most deadly cancers, and there have been few advances in treatment in the past decades [1]. Pancreatic cancer hardly responds to chemotherapy or immunotherapy, and surgery is usually not an option in most patients when diagnosed [2]. This situation is probably due to the special tumor microenvironment of pancreatic cancer. Unlike most other solid cancers, pancreatic cancer contains ample stromal cells, lacks vascularization, and is companied by persistent hypoxia within the tumor [3]. Hypoxia has been reported to induce aggressive characteristics of cancer cells in many types of cancer [4]. Therefore, pancreatic cancer is assumed to survive in long-term hypoxic conditions via special, but as yet unidentified, mechanisms.

Hypoxia can induce pancreatic cancer cells to undergo epithelial-to-mesenchymal transition (EMT) via several mechanisms, in which hypoxia-inducible factor 1α (hif-1α) always plays a pivotal role [5, 6]. Overexpression of hif-1α correlates with lymph node metastasis and poor prognosis in patients with pancreatic cancer [7]. However, studies suggested that hif-1α is mainly induced in severe hypoxia to help cells resist temporary stress [8, 9]. In addition, hypoxia can induce angiogenesis to relieve hypoxia, which is supposed to result in a persistently moderate or recurrently unstable hypoxic microenvironment within tumors [10]. Hif-2α, a homologous protein of hif-1α, was found to be more sensitive to moderate hypoxia and showed more enduring expression in hypoxic conditions [11]. Unfortunately, the roles of hif-2α in cancer are largely unknown, and the little evidence that exists is controversial. For instance, some studies showed that overexpression of hif-2α correlated with a poor prognosis [12], while others hypothesized that hif-2α was an indicator of good outcomes [13]. Similarly, the roles of hif-2α in pancreatic cancer are also under debate given the current limited data [14–16], and thus require further investigation.

β-Catenin is a crucial protein determines the fate of cancer cells, and has diverse functions. Wnt/β-catenin and hypoxia signaling have complex crosstalk between them [17, 18]. Previously, we confirmed that the interaction between hif-1α and β-catenin facilitated hif-1α-mediated EMT in liver cancer [19]. The interaction of hif-2α and β-catenin has also been reported in renal cancer [20]. However, the domains involved in the interactions between β-catenin and hif-αs were distinct, leading to opposite effects on classic Wnt/β-catenin signaling [20, 21]. In additional, complicated regulation of β-catenin by hif-2α was identified and is critical for early pancreatic tumorigenesis [22]. However, the effects of the interaction between hif-2α and β-catenin on pancreatic cancer have not been studied. Herein, we studied the effects of hif-2α in pancreatic cancer systematically, using cell lines, mouse models and human tissues. We also assessed the mutual influences between β-catenin and hif-2α signaling and its influence on tumor biology.

## Methods

### Cell culture and reagents

Pancreatic ductal adenocarcinoma (PDAC) cell lines (PANC-1, BxPC-3, SW 1990 and MIA PaCa-2) and human umbilical vein endothelium endothelial cell (HUVEC) were obtained from the Shanghai Institute for Biological Science (Shanghai, China). BxPC-3 and HUVEC were cultured in Roswell Park Memorial Institute (RPMI)-1640 Medium (GE Healthcare Life Science, Logan, UT, USA). PANC-1, SW 1990, and MIA PaCa-2 were cultured in high glucose Dulbecco’s modified Eagle’s medium (DMEM; HyClone, Logan, UT, USA). Both media were supplemented with 10% fetal bovine serum (FBS; Thermo Fisher Scientific, Waltham, MA, USA) and 1% penicillin/streptomycin (Genom, Zhejiang, China). All cells were maintained at 37.0 ± 0.2 °C in a humidified incubator with 5.0% CO_2_. Before exposure to the hypoxic environment (1.0% O_2_), cells were seeded in normoxic conditions and grown to approximately 60% confluence, or as specifically needed. After 24 h of serum-free culture, cells were exposed to a low-oxygen environment in a hypoxic chamber (Thermal Tech, Orlando, FL, USA) for the indicated duration.

Recombinant human Wnt3a was purchased from Abcam (Cambridge, MA, USA), and used at 200 ng/ml for 48 h. Sodium Chloride (NaCl) and Lithium Chloride (LiCl) was purchased from Sigma-Aldrich (St. Louis, MO, USA), and was used at 20 or 25 mM for 48 h. Recombinant human vascular endothelial growth factor (VEGF) was purchased from Peprotech (100-20A; Rocky Hill, NJ, USA), and was used at 20 ng/ml for 48 h. Desferrioxamine (DFO) was purchased from Sigma-Aldrich, and used at 100 μM for 24 h. The hif-2α translation inhibitor (CAS 882268–69-1) was purchased from Millipore (Darmstadt, Germany). Hif-2α antagonist (CAS 14422955–31-4) was purchased from Sigma-Aldrich. Cycloheximide (CHX; CAS 66–81-9) was purchased from Tocris Bioscience (Bristol, UK).

### Cell viability and 5-ethynyl-2′-deoxyuridine (EdU) assays

Before either the Cell Counting Kit-8 (CCK-8) or the EdU procedure, cells were seeded in 96-well plates at a density of 3000 cells per well for specific treatment. Cell viability was assessed using CCK-8 (Dojindo, Kumamoto, Japan). Cells were incubated with CCK-8 working solution for 3 h, after which the absorbance was measured at 450 nm using an ELx808 microplate reader (BioTeck). Relative cell viability was expressed as a proportion of specific controls.

The EdU Apollo 488 In Vitro Kit was purchased from Ribobio (Guangzhou, China) to examine cell proliferation. Medium supplemented with 20 μM EdU was added to the cells, followed by incubation for 2 h at 37 °C under 5.0% CO_2_. After fixation and neutralization according to the manufacturer’s instructions, Apollo and Hoechst 33,342 staining were performed, followed by observation under an inversion fluorescence microscope (Olympus IX51 Microsystems, Japan). The percentage of EdU-positive cells was calculated from five random fields in three wells.

### Transwell assays

The invasion activity of tumor cells was evaluated by their ability to pass through a gel matrix (Matrigel; BD, Franklin Lakes, NJ, USA). Briefly, Matrigel solution was diluted with FBS-free medium at a proportion of 1:5 to coat the 6.5 mm diameter polycarbonate filters (8 μm pore) of the Transwell chambers (Corning, NY, USA) in 24-well plates. With 40 ml working solution in every filter, the plates were placed at 37 °C for more than 5 h to solidify. Tumor cells were seeded at a density of 2 × 10^5^ per chamber and cultured with FBS-free medium in the upper compartments of the chamber for 48 h while the lower compartment was filled with complete medium. Non-invasive cells on the upper surface of the filter were wiped off using a cotton swab, while the cells adhering to the lower surface were fixed using 4% polyphosphate formaldehyde (Beyotime, Shanghai, China). These cells with stronger invasion potential were counted after crystal violet staining (Beyotime). The experiments were repeated for three times independently.

### Transfection, siRNA and luciferase reporter assays

Transfections were performed using Lipofectamine 3000 (Invitrogen, Carlsbad, CA, USA) according to the manufacturer’s instructions. All experiments were performed after the transfection medium was replaced by complete medium after 24 h of treatment. The hif-2α overexpression plasmid with the G to A point mutation (p.A530T) was a gift from Dr. Zhengping Zhuang from the National Institutes of Health (NIH; Bethesda, MD, USA).

Hif-1α siRNA (Thermo Fisher Scientific), Hif-2α siRNA (Santa Cruz Biotechnology, Santa Cruz, CA, USA), β-catenin siRNA (Cell Signaling Technology, Danvers, MA, USA) and negative-control siRNA (nc-siRNA; Santa Cruz) were transfected into cells at 100 nM. Luciferase reporter assays were carried out in 24-well plates, and 100 ng of TOPFlashplasmid, FOPFlashplasmid (Millipore) or hypoxia response element (HRE) reporter plasmids were also transfected. The HRE reporter plasmid was a gift from BM Emerling (Addgene plasmid #26731). Reporter activity was evaluated using the dual luciferase reporter system (Promega, Madison, WI, USA).

### Immunoblotting, co-immunoprecipitation, immunofluorescence and immunohistochemistry

These experiments were performed as described previously [19]. The following primary antibodies were from Cell Signaling Technology and were used at a 1:1000 dilution: anti-β-catenin, anti-E-cadherin, anti-vimentin, anti-Slug, anti-Snail, anti-Oct-4, anti-Sox2, anti-Nanog, and anti-glyceraldehyde-3-phosphate dehydrogenase (GAPDH). Other primary antibodies included anti-hif-1α (1:500; Abcam), anti-hif-2α (1:500; Abcam). The secondary antibodies were goat anti-rabbit antibodies conjugated with horseradish peroxidase (1:2000; Cell Signaling Technology). For the CHX test, Image J software (NIH) was used to evaluate the photodensity of immunoblotting bands.

For the co-immunoprecipitation experiments, about 300 μg of protein extract supplemented with 1 μg of specific primary antibody or an isogenic control antibody, was incubated for 4 h at 4 °C and then for another 2 h after 10 μl of pre-rinsed protein-A/G sepharose (Santa Cruz) was added. The samples were then centrifuged (1000 g), rinsed, and formulated as a sample by adding 1× loading buffer for immunoblotting and analysis.

For immunofluorescence staining, cells were fixed with cold 4% polyphosphate formaldehyde for 15 min and washed with PBS. Permeabilization was then performed with 1% Triton for over 30 min, followed by blocking with 5% bovine serum albumin (BSA) for 30 min at room temperature. After washing, the cells were co-incubated with anti-β-catenin mouse antibody (1:200; Cell Signaling Technology) and anti-hif-2α rabbit antibody (1:100; Abcam) at 4 °C overnight, and were further incubated with the corresponding secondary antibodies conjugated to different fluorescent dyes for 30 min in the dark at room temperature. Counterstaining with 4′,6-diamidino-2-phenylindole (DAPI; 1:10,000; Sigma) was carried out 3–5 min before observation under an inversion fluorescence microscope (Olympus Optical, Tokyo, Japan). Stained samples without primary antibody were used as negative controls.

For immunohistochemical staining, formalin-fixed, paraffin-embedded PDAC tissue samples were cut into 5-μm thick serial sections which were then incubated with anti-hif-1α (1:100), anti-hif-2α (1:100), anti-β-catenin (1:100), anti-E-cadherin (1:100) or anti-vimentin (1:100) antibodies, respectively. The slides were then incubated with HRP-conjugated antibodies against rabbit IgG using Histostain-Plus Kit (ZSGB-BIO, Beijing, China). The sections were counterstained with hematoxylin, and the slides were inspected under a microscope (Leica, Heidelberg, Germany). Negative controls were incubated with PBS instead of the specific primary antibodies.

### Enzyme-linked immunosorbent assay (ELISA)

The supernatants were collected and stored at −80 °C. Concentrations of matrix metalloproteinase-9 (MMP-9) and VEGF in the conditioned media were detected using ELISA kits (R&D Systems, Minneapolis, MN, USA) according to the manufacturer’s instructions.

### Flow cytometry

After specific treatment, pancreatic cancer cells were stained with fluorochrome-conjugated monoclonal antibodies for CD133 (Miltenyi Biotec, Teterow, Germany) or an isotype control antibody (Miltenyi Biotec). Apoptosis assay was performed using the Annexin V FITC Apoptosis Detection KIT (BD Biosciences). Flow cytometric analysis was performed on a BD FACSCanto II system (BD Biosciences).

### Mouse models

The Ethics Committee of the Second Affiliated Hospital of Zhejiang University School of Medicine (SAHZJU) approved the study protocol for the use of experimental animals. Male *Balb/c nude* mice (18–22 g) were purchased from Shanghai Experimental Animal Center (Shanghai, China). To assess role of hif-2α in tumor progression, each mouse was injected subcutaneously with 250,000 treated PANC-1 or SW 1990 cells suspended in 200 μL of medium. Mice were sacrificed after 6 weeks, and the xenograft volume was monitored by weight. Pathological scores were evaluated independently by a pathologist, according to the expression degrees of indicated proteins as described previously [19].

### Metabolic phenotype assessment

The basal metabolic level and metabolic phenotype were detected by using a Seahorse XFe96 Analyzer (Seahorse Bioscience, North Billerica, MA, USA). PANC-1 cells were seeded in a 6-well plate, and transfected with pcDNA3 or the hif-2α overexpression plasmid. After 48 h, cells were seeded at 2,5000 per well with eight wells per group for the experiments. Stress assessment was performed using 10 μM of oligomycin and 20 μM of Carbonyl cyanide 4-(trifluoromethoxy)phenylhydrazone (FCCP). Experiments were performed according to the instructions of the XF cell energy phenotype test kit (Seahorse Bioscience).

### Acquisition of human tissue

Formalin-fixed, paraffin-embedded PDAC tissue samples were obtained from the SAHZJU. All the patients with PDAC underwent curative resection between 2010 and 2015, and samples from these patients were used for immunochemistry analysis. This project was approved by the Ethics Committee of the SAHZJU.

### Statistical analysis

Data are presented as the mean ± standard deviation (SD) or standard error of the mean (SEM), as appropriate. Statistical calculations were performed using Prism 6 software (GraphPad, San Diego, CA, USA), or as otherwise indicated. Statistical analyses were performed using the *F* test following a two-tailed unpaired Student’s *t*-test, except for in vivo data, which were analyzed using a paired Student’s *t*-test. Protein half-life curves were generated using a one-phase exponential decay model. Best-fit lines and their 95% confidential intervals for immunochemistry results of xenografts were calculated using linear regression. Survival data were analyzed using Kaplan-Meier curves and a log-rank test. Univariate and multivariate analyses were performed using SPSS software (v22.0, IBM Corp, Bethesda, MD, USA). For all tests, a *P* value less than 0.05 was considered statistically significant.

## Results

### Hif-2α was associated with hypoxia-induced EMT in pancreatic cancer

**Fig. 1:**
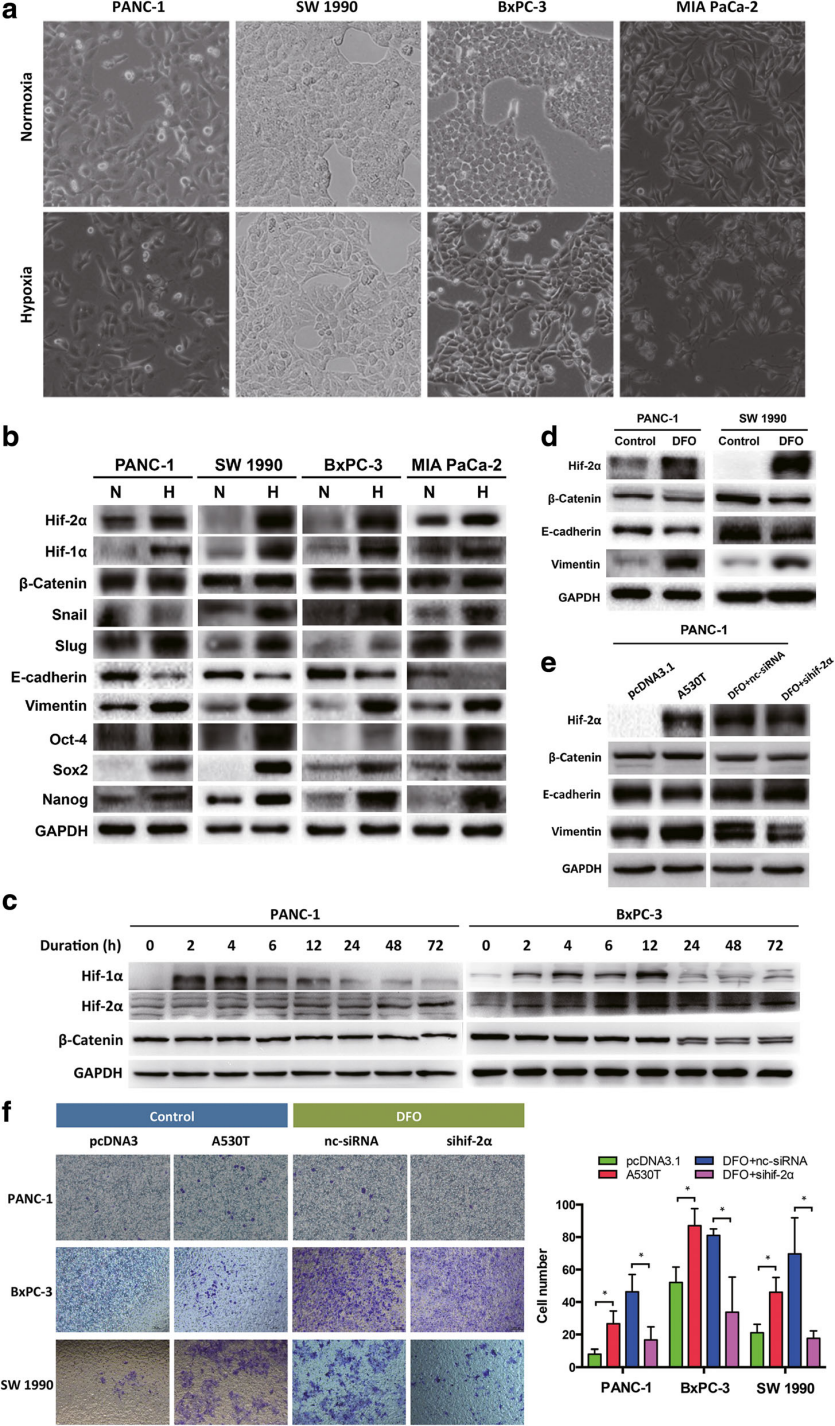
Hif-2α mediated hypoxia induced EMT in pancreatic cancer. **a** Morphology changes of PDAC cells after undergoing hypoxia for 48 h. **b** PDAC cells underwent hypoxia showed expression changes of hypoxia-related (hif-1α, hif-2α), EMT-related (β-catenin, Snail, Slug, E-cadherin, vimentin), and stemness-related (Oct-4, Sox2, Nanog) genes. **c** Early and late accumulation of hif-α proteins after different duration of hypoxia. **d** DFO mimicked hypoxia and induced EMT in PANC-1 and SW 1990 cells. **e** Regulation of hif-2α level changed vimentin expression in PANC-1 cells. **f** Overexpression of degradation-resistant hif-2α (A530T) increased invasion capacities of the PDAC cells, and inhibition of hif-2α decreased their invasion abilities. *, *P* < 0.05 as compared to pcDNA3 or DFO + nc-siRNA

Initially, we used our previous model to mimic hypoxia-induced EMT in four pancreatic cancer cell lines [19]. As expected, hypoxia induced morphological changes of the cells, especially BxPC-3 and SW 1990 cells (Fig. 1a), accompanied by decreased expression of E-cadherin and increased expression of vimentin (Fig. 1b). However, when tracing the expressions of hif-1α and hif-2α during hypoxia, we noticed that hif-1α rapidly increased and peaked before 12 h after initiation of hypoxia, while the protein level of hif-2α gradually increased up to 72 h after initiation of hypoxia (Fig. 1c). DFO was also used to mimic hypoxia, and overexpression of hif-2α and down-regulated E-cadherin and up-regulated vimentin were observed (Fig. 1d). Although β-catenin was reported to mediate EMT in pancreatic cancer [23, 24], we detected either no change or even a trend of down-regulation of β-catenin following hypoxia (Fig. 1c and d).

To investigate the specific role of hif-2α in pancreatic cancer, we excluded the influence of hif-1α by selectively modifying hif-2α expression. Plasmids encoding a stabilized type of hif-2α (A530T), which is resistant to prolyl hydroxylase-dependent degradation [25], was used to up-regulate hif-2α. In PANC-1 cells, overexpression of hif-2α significantly enhanced the expression of vimentin, while inhibition of hif-2α by a short interfering RNA (siRNA) induced a reduced level of vimentin in hypoxic conditions (Fig. 1e). Consistently, the cell migration ability was increased by hif-2α overexpression and reduced by hif-2α interference (Fig. 1f). These results implicated that hif-2α was able to regulate EMT in pancreatic cancer.

### Activity of β-catenin was up-regulated by hif-2α

**Fig. 2:**
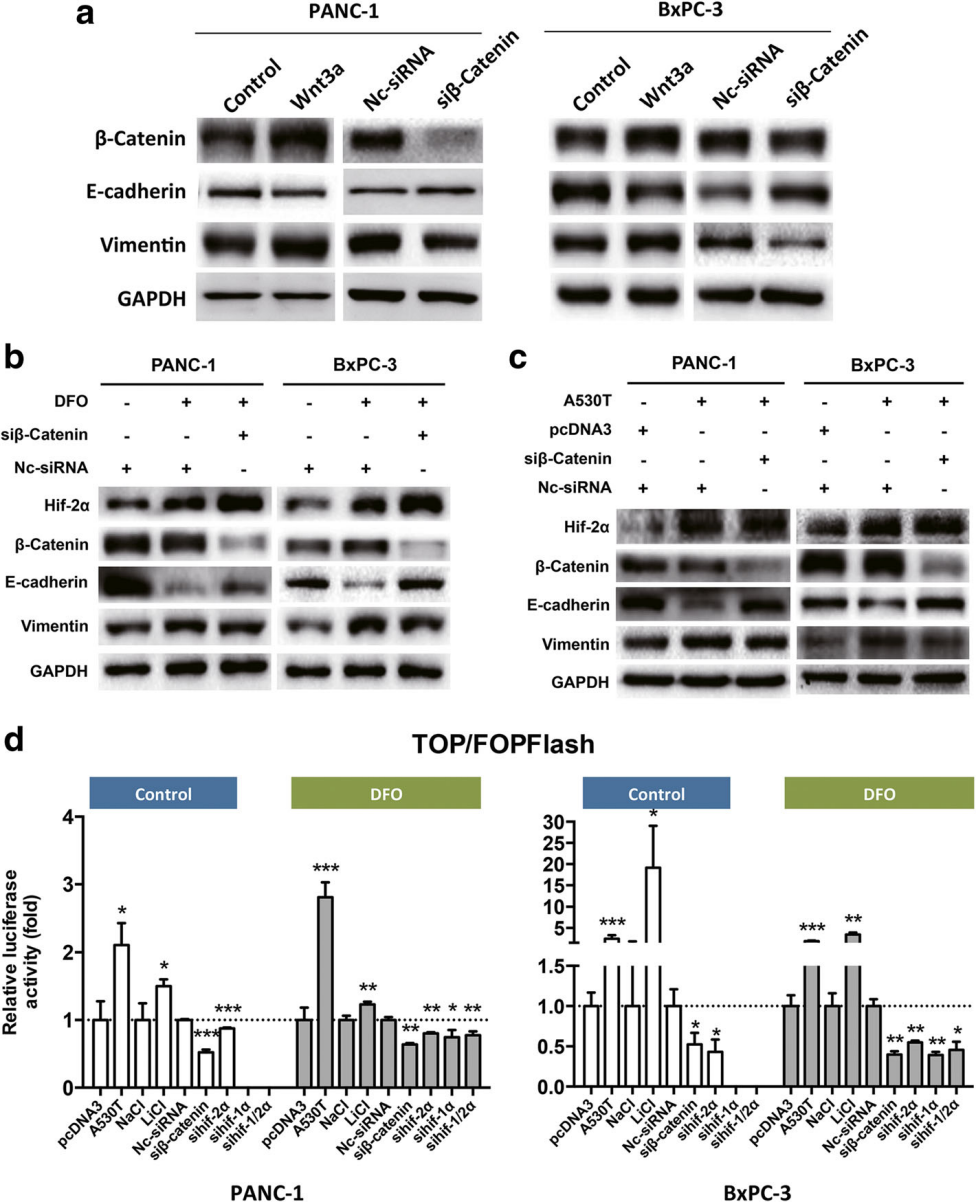
Hif-2α enhanced the activity of β-catenin. **a** Wnt-3a induced β-catenin promoted EMT while siRNA inhibited β-catenin blocked EMT in PANC-1 cells. **b** DFO promoted EMT, which was partially reversed by β-catenin interference by siRNA. **c** Hif-2α (A530T) promoted EMT, which was partially reversed by β-catenin interference by siRNA. **d** Hif-2α (A530T) transfection enhanced transcription activity of β-catenin in PANC-1 and SW 1990 cells regardless of oxygen availability. Knockdown of hif-2α reduced β-catenin activity as measured by luciferase reporter assays. *, *P* < 0.05; **, *P* < 0.01; ***, *P* < 0.001, as compared to pcDNA3, NaCl, or nc-siRNA. Representatives of three independent experiments

Consistent with previous reports [23, 24], β-catenin was sufficient to induce EMT in pancreatic cancer. Up-regulation of β-catenin by Wnt3a was associated with increased expression of vimentin and reduced expression of E-cadherin, while inhibition of β-catenin using siRNA led to the opposite results (Fig. 2a). However, hif-2α-mediated EMT in pancreatic cancer was abolished when β-catenin expression was inhibited (Fig. 2b and c), suggesting that hif-2α regulated EMT in a β-catenin-dependent manner.

Given that neither hypoxia nor hif-2α overexpression increased the level of β-catenin in our conditions (Figs. 1c, d, and 2c), we speculated that hif-2α might regulate the transcriptional activity of β-catenin. Indeed, hif-2α positively regulated β-catenin activity in PANC-1 and BxPC-3 cells cultured in both normal and hypoxic conditions (Fig. 2d). Taken together, these results suggested that regulation of β-catenin activity was responsible for hif-2α-mediated EMT in pancreatic cancer.

### β-catenin reduced degradation of hif-2α and its transcriptional activity

**Fig. 3:**
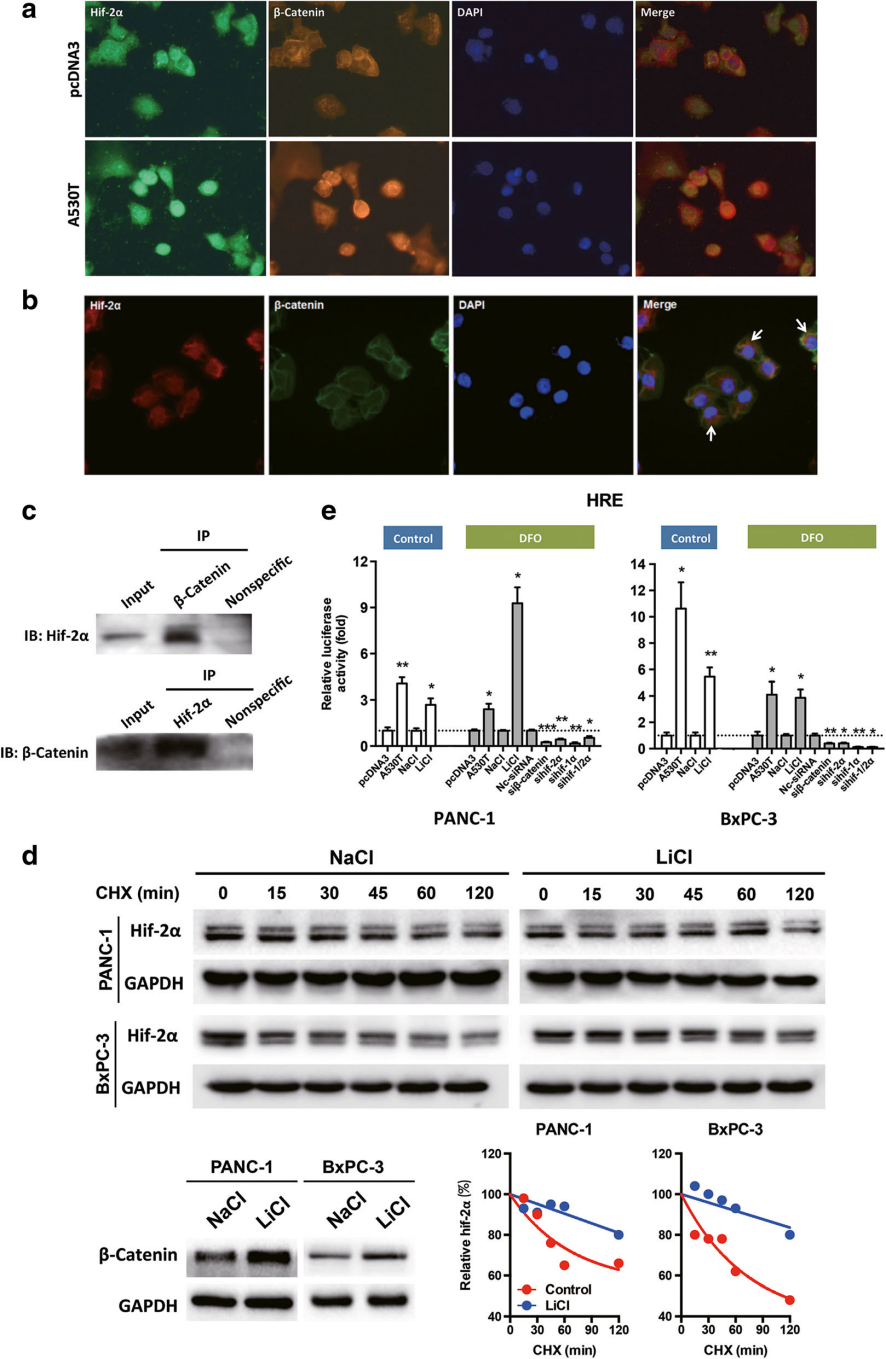
Complex formation of hif-2α/β-catenin and its influence on each other. **a** Overexpression of hif-2α induced nuclear translocation of β-catenin in PANC-1 cells. **b** Co-existence of hif-2α and β-catenin in PANC-1 cells. **c** Interaction of hif-2α and β-catenin was noticed in PANC-1 cells. **d** The half-life of hif-2α was increased when β-catenin was up-regulated by LiCl. Cycloheximide was used to block protein biosynthesis. Densitometry was used to compare the degradation degree of the protein. **e** Increased β-catenin expression by LiCl enhanced HRE activity, and knockdown of β-catenin decreased HRE activity in normoxic or hypoxic conditions as indicated. *, *P* < 0.05; **, *P* < 0.01; ***, *P* < 0.001, as compared to pcDNA3, NaCl, or nc-siRNA. Representatives of two independent experiments

The co-existence of hif-2α and β-catenin was observed in pancreatic cancer cells, especially those with hif-2α overexpression, which resulted in nuclear translocation of β-catenin (Fig. 3a and b), suggesting that the transcriptional activity-dependent role of β-catenin was facilitated by hif-2α in these cells. Co-immunoprecipitation confirmed the physical interaction between hif-2α and β-catenin (Fig. 3c). Intriguingly, overexpression of β-catenin improved the stability of endogenous hif-2α protein by significantly increasing its half-life, resulting in a higher level of hif-2α within the cells (Fig. 3d). Correspondingly, up-regulation of β-catenin by LiCl elevated HRE activity, whereas decreased HRE activity was found when β-catenin was inhibited by siRNA (Fig. 3e). These findings suggested that a high level of β-catenin maintained the abundance and activity of hif-2α in pancreatic cancer.

### Hif-2α increased the proliferation, metabolic shift, and stemness of pancreatic cancer cells

**Fig. 4:**
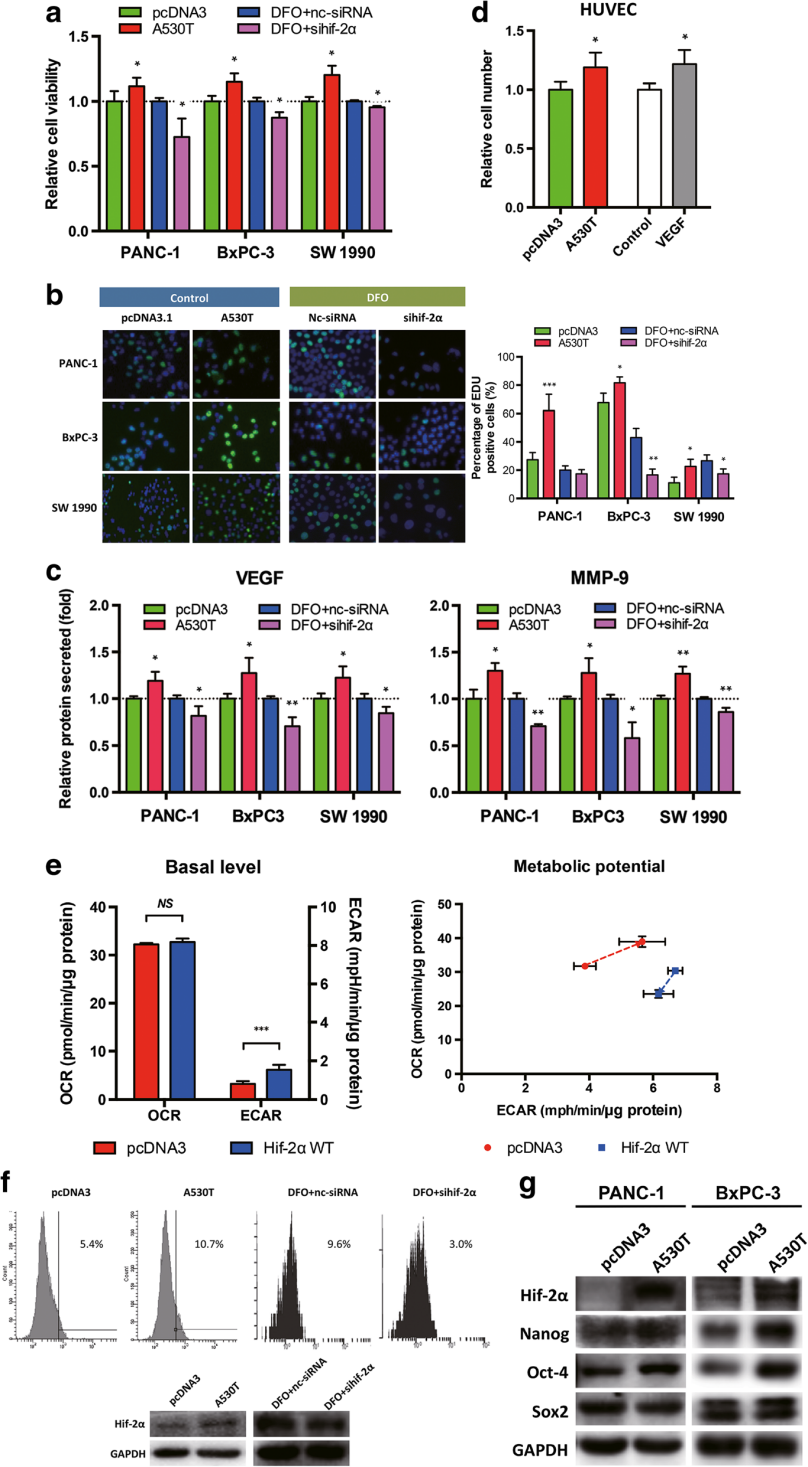
Hif-2α promoted malignant characteristics of pancreatic cancer cells. **a-b** Overexpression of hif-2α enhanced cell viability and proliferation ability of PDAC cells, while hif-2α knockdown decreased the viability and proliferation ability of cells exposed to DFO. **c** Hif-2α positively regulated secretion of PDAC cell-derived MMP-9 and VEGF. *, *P* < 0.05; **, *P* < 0.01; ***, *P* < 0.001, as compared to pcDNA3 or DFO + nc-siRNA. **d** Hif-2α overexpression increased proliferation of HUVECs. VEGF was used as the positive control. *, *P* < 0.05, as compared to pcDNA3 or vehicle control. **e** PANC-1 cells with hif-2α overexpression showed higher activity of aerobic glycolysis but without any change in oxidative phosphorylation (left panel). The cells also had limited glycolysis potential under stress (right panel). ***, *P* < 0.001, as compared to pcDNA3. **f** Up-regulation of hif-2α increased the number of CD133^+^ PANC-1 cells. Down-regulation of hif-2α inhibited DFO-induced increase of CD133^+^ cells. **g** Overexpression of hif-2α up-regulate expression of Oct-4 and Nanog in PANC-1 and BxPC-3 cells

β-Catenin is closely associated with cell proliferation and stemness; therefore, we tested whether hif-2α affected these characteristics of pancreatic cancer. In all three cell lines tested, overexpression of stabilized hif-2α significantly improved cell viability, which was inhibited by hif-2α interference in DFO-mimicked hypoxia (Fig. 4a). The same effects of hif-2α were also found in EdU assays (Fig. 4b), suggesting that hif-2α could enhance pancreatic cancer cell proliferation. However, hif-2α had no effect on apoptosis of these malignant cells (data not shown). Furthermore, as target genes of hif-2α [26, 27], secretion of VEGF and MMP9 were both enhanced by hif-2α overexpression and inhibited by hif-2α knockdown (Fig. 4c). This paracrine signaling might stimulate vascularization and support tumor cell proliferation, as indicated by increased proliferation of HUVEC cells (Fig. 4d).

Cancer cells tend to shift from oxidative phosphorylation to glycolysis even when sufficient oxygen is available, which is termed the “Warburg effect” [28]. Cancer cells are believed to benefit from this metabolic shift by acquiring sufficient biosynthetic capability for their proliferation. We observed that overexpression of hif-2α elevated aerobic glycolysis without significant changes in mitochondrial respiration (Fig. 4e, left panel). Notably, hif-2α overexpression changed the metabolic potential of these cells profoundly (Fig. 4e, right panel). In addition, overexpression of hif-2α increased the proportion of CD133 positive cells (Fig. 4f). Correspondingly, stemness-associated proteins Nanog and Oct-4 were up-regulated when hif-2α was highly expressed, although the level of Sox2 was unchanged (Fig. 4g). These results suggested a possible role of hif-2α in maintaining the stemness of certain cancer stem cells.

### Hif-2α promoted tumor growth and EMT in mouse models

**Fig. 5:**
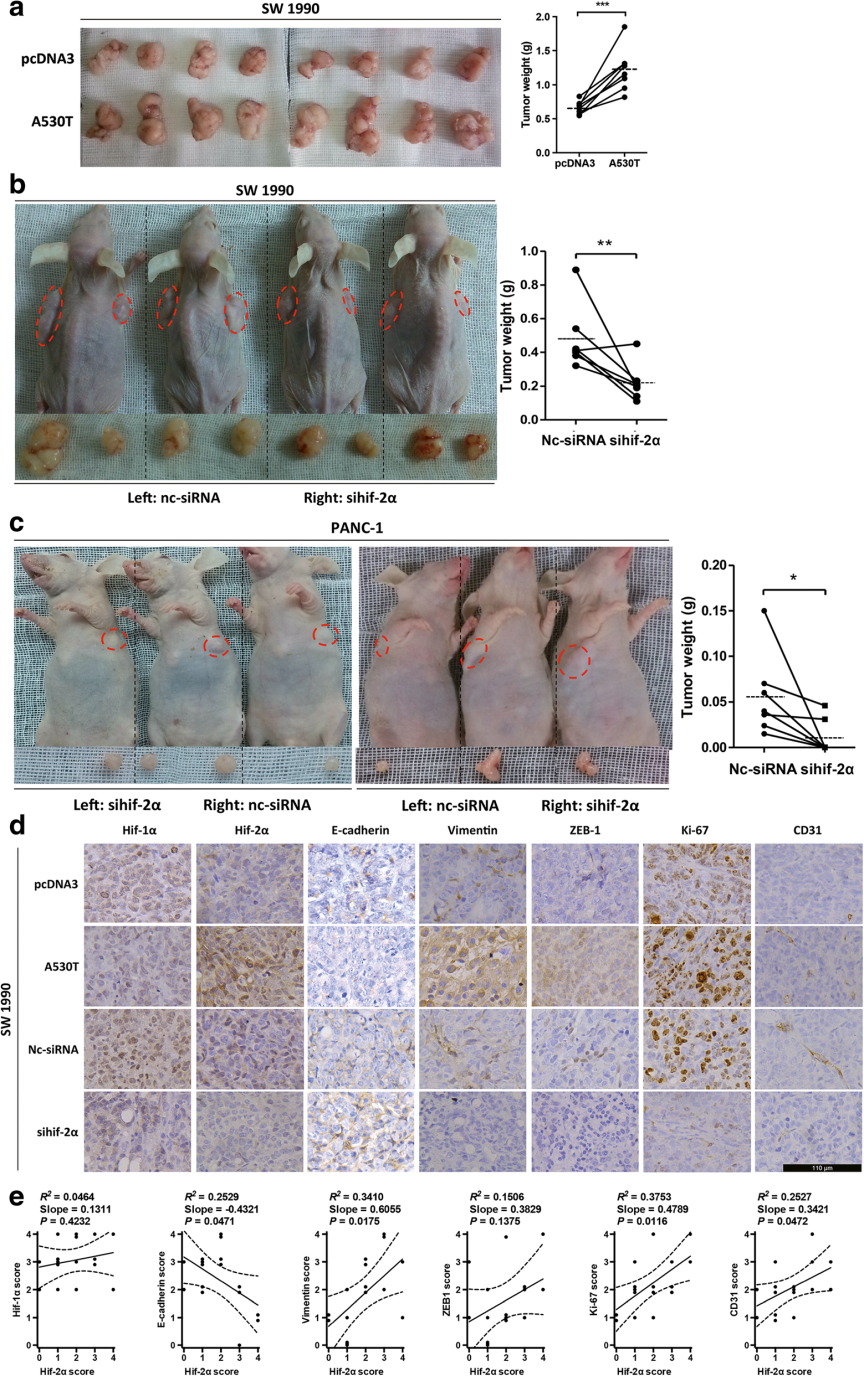
Hif-2α promote tumor progression in mouse models. **a** SW 1990 cells transfected with pcDNA3 or hif-2α (A530T) were injected subcutaneously as pairs in mice. The xenografts were much bigger when hif-2α (A530T) was transfected. ***, *P* < 0.001. **b** Nc-siRNA or sihif-2α was transfected to SW 1990 cells and a similar mouse model with a was generated. The xenografts with sihif-2α weighted lighter than nc-siRNA controls. **, *P* < 0.01. Dash lines indicate averages of tumor weight. **c** A similar mouse model with b was performed for PANC-1 cells instead of SW 1990 cells. Hif-2α knockdown inhibited xenograft growth. *, *P* < 0.05. Dash lines indicate averages of tumor weight. **d** Sixteen xenografts from mice in a and b were harvested and immunochemistry was performed for several proteins. Expression of vimentin, ZEB-1, Ki-67, and CD31 was positively correlated with hif-2α, while E-cadherin expression was negatively correlated with hif-2α. Bar, 110 μm. **e** Correlation analyses of expression between hif-2α and hif-1α, E-cadherin, vimentin, ZEB-1, Ki67, and CD31

To confirm the role of hif-2α in vivo, we first used an SW 1990 xenograft mouse model. Overexpression of stabilized hif-2α promoted tumor growth, with significantly higher tumor weights (Fig. 5a). Inhibition of hif-2α using siRNA was associated with decreased tumor growth in the same mouse model (Fig. 5b). In parallel, a similar result was found when the PANC-1 xenograft mouse model was used (Fig. 5c). We then analyzed the expressions of certain proteins in the xenograft tissue. Hif-2α was positively correlated with the expression of vimentin, Ki-67, CD31, and negatively correlated with the expression of E-cadherin (Fig. 5d and e), indicating that hif-2α facilitated EMT and promoted tumor growth in the mouse model. Intriguingly, unlike hif-2α, the level of hif-1α was relatively high in all xenografts, and no correlation was detected between hif-2α and hif-1α (Fig. 5e), suggesting that other mechanisms of hif-2α induction exist besides hypoxia.

### Overexpression of hif-2α correlated with poor prognosis in patients

**Fig. 6:**
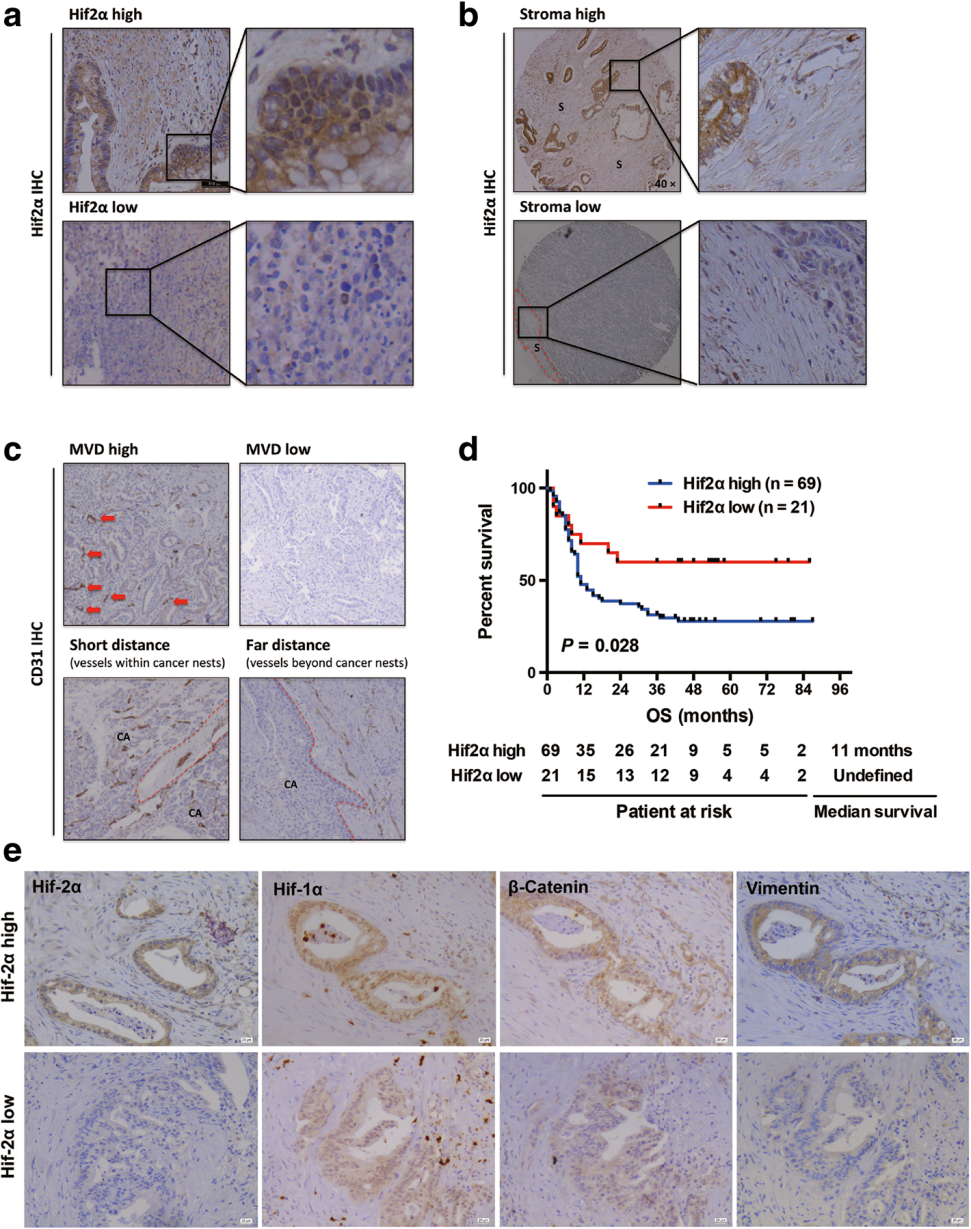
Hif-2α was associated with poor prognosis in patients with pancreatic cancer. **a-c** In pancreatic cancer tissue array, overexpression of hif-2α was correlated with abundant stroma, high microvascular density within tumors as well as short tumor-vessel distance. **d** Patients with high expression of hif-2α had significantly shorter survival compared to those with low hif-2α expression. **e** Representatives of successive series of PDAC tissue showed overexpression of vimentin in tumors with high hif-2α expression

We tested hif-2α expression and its clinical relevance in human pancreatic cancer tissue. Hif-2α overexpression was detected in around 70% of samples, and was significantly correlated with high stroma abundance, high tumor grade, and short distance between vessels and tumor cells (Table 1, Fig. 6a-c). A borderline increased risk of lymph node metastasis was also found (*P* = 0.052). Importantly, high expression of hif-2α could be an independent factor for prognosis with a hazard ratio of 2.7 (Table 2). Patients with pancreatic cancer had much better overall survival if low expression of hif-2α was confirmed, although the proportion of such patients was relatively low (Fig. 6d). Additionally, several successive sections were further checked to identify the co-existence of certain proteins. High expression of hif-2α was correlated with high levels of β-catenin and vimentin, without significant differences in hif-1α expression (Fig. 6e).

**Table 1:** Correlation between hif2α expression and clinicopathological information in 90 pancreatic cancer patients

	Hif-2α low	Hif-2α high	*P* value
Lymph node metastasis^a^			0.052
No	16	35	
Yes	5	28	
Stroma abundance			< 0.001
Low	8	5	
High	13	64	
Pathological grade^b^			0.011
I/II	13	55	
III/IV	7	5	
Vascular distance^c^			0.006
Far	13	18	
Short	7	41	

^a^Six patients were excluded due to absence of relative data

^b^Ten patients were excluded due to absence of relative data

^c^Eleven patients were excluded due to absence of relative data

**Table 2:** Univariate and multivariate analyses indicated factors for patient survival

Variable	Univariate analysis		Multivariate analysis	
	HR	*P* value	HR (95% CI)	*P* value
Age (≥ 60/< 60)	1.057	0.833	-	
Gender (male/female)	1.907	0.025	1.582 (0.882–2.839)	0.124
Disease stage (1/2)	0.568	0.033	0.661 (0.384–1.136)	0.134
Hif-2α (high/low)	2.214	0.028	2.693 (1.189–6.097)	0.018
Stroma (high/low)	1.221	0.585	-	
TNM stage (I + II/III + IV)	0.638	0.207	-	
Microvascular density (high/low)	0.977	0.937	-	
Tumor location (head/body or tail)	0.851	0.463	-	
Nerve invasion (yes/no)	1.239	0.411	-	

## Discussion

Pancreatic cancer is characterized by poor vascularization. However, unlike other solid tumors, pancreatic cancer cells undergo persistent hypoxia because of the highly proliferated stromal cells and abundant extracellular matrix [29]. Hif-1α-mediated hypoxia adaption has been used frequently to explain the survival advantage of cancer cells under hypoxic conditions [7, 30]. Unfortunately, hif-1α behaves more like a stress response protein to protect cells against acute threats. Although hif-1α is accumulated in the tumor microenvironment, it is probably a passive phenomenon and is not the main mechanism responsible for chronic or persistent hypoxia. Consistent with previous studies [11, 31], we found that hif-2α, rather than hif-1α, showed a chronic response pattern, which was more compatible with the observed chronic metabolic changes of cancer cells in continuous hypoxia [32]. Here, we identified several roles of hif-2α in pancreatic cancer, and in particular, investigated its crosstalk with β-catenin.

Hif-2α is highly homologous with hif-1α, and their target genes largely overlap. However, distinct or even opposing roles of the two proteins in tumor progression have been reported recently [33]. Although hif-2α was considered as a pro-tumor factor in digestive system cancers including pancreatic cancer [14, 16, 34], some investigators reported a better prognosis in pancreatic cancer patients with hif-2α overexpression [15]. Thus, the functional effects of hif-2α in pancreatic cancer require in-depth study. Unfortunately, it is difficult to differentiate the specific effects of hif-2α from those of hif-1α because both proteins can be easily accumulated under hypoxic conditions. In addition, hif-2α is sensitive to oxygen and is quickly degraded, making traditional overexpression strategies difficult. In this study, we used an A530T mutated hif-2α whose function was not impaired but whose half-life was three times longer than that of wild-type hif-2α [25]. Although no such mutation on hif-2α has been reported in pancreatic cancer, pancreatic cancer cells harbor a high level of hif-2α protein [16] because of the special tumor microenvironment that is difficult to mimic in vitro. Our strategy succeeded in highlighting the roles of hif-2α in not only proliferation and migration, but also in cancer stem cell renewal and angiogenesis.

The interaction between hif-2α and β-catenin has been noticed in renal carcinoma [20]. Here, we confirmed the hif-2α/β-catenin complex formation in pancreatic cancer. However, hif-1α can also interact with β-catenin [19], making the interactions among these three proteins extremely complicated. The hif-1α/β-catenin complex increases the transcriptional activity of hif-1α [21], while hif-2α/β-catenin enhances β-catenin transcription [20]. However, interaction between hif-1α and β-catenin compromised β-catenin activity [19–21], which was improved by the interaction between hif-2α and β-catenin, as confirmed here and in a previous study [20]. The domains of β-catenin responsible for interaction with the two hif-αs are different. Although both hif-αs use their N-termini (aa 1–344 in hif-1α [21] and aa 1–67 in hif-2α [20]) to bind β-catenin, the C-terminus (aa 530–781^20^ or aa 531–722^21^ according to different studies) and N-terminus (aa 1–259 [20]) of β-catenin are involved in the interactions with hif-1α and hif-2α, respectively [20, 21]. The detection of hif-1α/β-catenin/hif-2α complex in pancreatic cancer (data not shown) also suggested a noncompetitive interaction between hif-1α and hif-2α for β-catenin binding.

Unexpectedly, we found that hif-2α/β-catenin could stabilize hif-2α and increase its transcriptional activity, although the mechanism was not identified. The N-terminal end, but not the oxygen-dependent degradation domain, of hif-2α interacts with β-catenin; we presumed that an untypical mechanism exists to block of hif-2α degradation, which requires further investigation. This finding, however, implied a positive-feedback regulation between hif-2α and β-catenin, which might be critical in carcinogenesis and the development of pancreatic cancer. In fact, Criscimanna et al. discovered that the mutual regulation of the two proteins plays an important role during early pancreatic tumorigenesis [22]. In their study, an early-stage decreased and late-stage increased pattern of hif-2α expression favored tumor initiation that developed from pancreatic intraepithelial neoplasia [22]. Therefore, the β-catenin-dependent stabilization of hif-2α might explain why the level of hif-2α increases in the late stage.

## Conclusions

Taken together, this study revealed a pro-tumor role of hif-2α in pancreatic cancer, both in vitro and in vivo. Hif-2α enhances tumor proliferation, invasion, stemness and angiogenesis. We found that hif-2α interacts with β-catenin, leading to elevated classic Wnt/β-catenin activity. Moreover, we also revealed that formation of the hif-2α/β-catenin complex stabilizes hif-2α and facilitates its transcriptional activity. Finally, we reported poor prognosis in patients with high hif-2α expression, making hif-2α a possible target for pancreatic cancer therapy.

## Abbreviations

*BSA*: Bovine serum albumin
*CCK8*: Cell Counting Kit-8
*CHX*: Cycloheximide
*DAPI*: 4′,6-diamidino-2-phenylindole
*DFO*: Desferrioxamine
*EdU*: 5-ethynyl-2′-deoxyuridine
*ELISA*: Enzyme-linked immunosorbent assay
*EMT*: Epithelial-to-mesenchymal transition
*FBS*: Fetal bovine serum
*FCCP*: Carbonyl cyanide 4-(trifluoromethoxy)phenylhydrazone
*GAPDH*: Glyceraldehyde-3-phosphate dehydrogenase
*hif-1*α: Hypoxia-inducible factor-1α
*hif-2*α: Hypoxia-inducible factor-2α
*HRE*: Hypoxia response element
*HUVEC*: Human umbilical vein endothelium endothelial cell
*LiCl*: Lithium chloride
*MMP-9*: Matrix metalloproteinase-9
*NaCl*: Sodium chloride
*PDAC*: Pancreatic ductal adenocarcinoma
*RPMI*: Roswell Park Memorial Institute
*siRNA*: Short interfering RNA
*VEGF*: Vascular endothelial growth factor
